# Changes of the Macular Ganglion Cell-Inner Plexiform Layer Thickness after Cataract Surgery in Glaucoma Patients

**DOI:** 10.1155/2016/9785939

**Published:** 2016-12-22

**Authors:** Hyun Cheol Roh, Choul Yong Park, Martha Kim

**Affiliations:** Department of Ophthalmology, Dongguk University Ilsan Hospital, Goyang, Republic of Korea

## Abstract

*Purpose*. To investigate the effect of uneventful cataract surgery on macular ganglion cell-inner plexiform layer (mGC-IPL) thickness in glaucoma patients.* Methods*. This retrospective study included 65 eyes of 65 subjects who underwent uneventful cataract surgery, including 13 glaucoma eyes and 52 normal eyes. Using spectral domain optical coherence tomography, the mGC-IPL thickness was measured and compared between glaucoma and normal eyes preoperatively as well as 1 month and 3 months postoperatively. Linear regression analysis was used to determine the factors associated with postoperative change in mGC-IPL thickness.* Results*. The mean mGC-IPL significantly increased in both groups 1 month and 3 months after surgery (all* P* values equal to or less than 0.001). The postoperative changes between groups were not significantly different (*P* = 0.171). In the multivariate regression analysis, preoperative mGC-IPL thickness showed a significant association with the change of average mGC-IPL thickness 1 month and 3 months after surgery (all* P* values < 0.001).* Conclusions*. The mean mGC-IPL thickness was increased after cataract surgery, and the postoperative mGC-IPL thickness changes were associated with preoperative mGC-IPL thickness in both groups and axial length in normal eye. The effects of cataract surgery on mean mGC-IPL thickness were not different in glaucomatous and normal eyes.

## 1. Introduction

Glaucoma is characterized by the loss of retinal ganglion cells (RGC) and their axons, which becomes clinically manifest in structural changes to the optic nerve head and retinal nerve fiber layer (RNFL) and in functional changes to visual fields. Recent studies have found that measuring macular ganglion cell complex (GCC) thickness provides diagnostic performance similar to RNFL thickness for glaucoma detection [1, 2]. GCC thickness consists of the three innermost retinal layers: RNFL, the ganglion cell layer, and the inner plexiform layer (IPL). Measuring RNFL and macular thickness, optical coherence tomography (OCT) is a useful tool for diagnosing glaucoma. In fact, high definition (HD) OCT provides a ganglion cell analysis (GCA) program able to measure the thickness of the macular ganglion cell-inner plexiform layer (mGC-IPL) [3]. Previous research has reported that the mGC-IPL thickness measurement and RNFL thickness measurement have a similar capacity for detecting glaucoma [4].

Cataract surgery is the most commonly performed operation in the field of ophthalmology, even in glaucoma patients. Several studies have investigated the effect of cataract surgery on macula and RNFL using different OCT platforms [5–11]. Recent studies have shown that the GCC and peripapillary RNFL thickness significantly increased after routine cataract surgery in nonglaucomatous eyes [12, 13]. To our knowledge, there have been few studies evaluating changes in mGC-IPL thickness after cataract surgery in glaucomatous eyes. To fill this gap, the current study aims to determine the effect of uneventful cataract surgery on mGC-IPL thickness change and to compare the change between glaucomatous and nonglaucomatous eyes.

## 2. Materials and Methods

Following the tenets of the Declaration of Helsinki, this retrospective clinical study was approved by the Institutional Review Board of the Dongguk University Ilsan Hospital. Patients who had undergone routine cataract surgery in one eye were recruited consecutively for this study. Between March 2012 and December 2013, a total of 65 eyes from 65 subjects were studied, including 13 eyes with glaucoma and 52 normal eyes. All glaucoma patients were required to have open angles and their intraocular pressure (IOP) was well controlled under medications.

All subjects included in this study underwent a complete preoperative ophthalmic examination that included the following tests: best-corrected visual acuity, measurement of IOP, slit lamp examination, dilated fundus examination, corneal thickness measurement by noncontact specular microscope (Konan Noncon Robo-8400, Konan Medical, Hyogo, Japan), axial length (AXL) measurement by IOL Master (Carl Zeiss Meditec, Dublin, CA, USA), and Cirrus HD OCT imaging (Carl Zeiss Meditec, Dublin, CA, USA). Patients with glaucomatous optic neuropathy also underwent an automated perimetry using a Humphrey Visual Field Analyzer (Carl Zeiss Meditec, Dublin, CA, USA).

Glaucoma subjects had glaucomatous optic disc change, RNFL defects with corresponding neuroretinal rim thinning, and glaucomatous visual field defects. This study included only patients with reliable perimetry results, defined as false-negative and false-positive responses < 33% and fixation loss < 20%. Glaucomatous visual field defect was determined according to Anderson's criteria [14] as follows: (1) having a cluster of 3 points with *P* < 5% in the pattern deviation plot in a single hemifield, including at least 1 point with *P* < 1%, (2) receiving glaucoma hemifield test results outside the normal limit, or (3) having a pattern standard deviation of < 5%.

Exclusion criteria included prior intraocular surgery, prior glaucoma surgery, or preexisting retinal pathologies, such as diabetic retinopathy, epiretinal membrane, or macular degeneration. Patients with cystoid macular edema after cataract surgery were also excluded. All the patients had no intraoperative complication such as posterior capsular rupture or vitreous prolapse.

### 2.1. Surgical Technique

A single surgeon (CYP) performed phacoemulsification and intraocular lens (IOL) insertion. Under topical anesthesia, a side-port incision was made with a 1 mm diamond knife; then, the main clear corneal incision was made with a 2.8 mm diamond knife. An ophthalmic viscosurgical device, 4% chondroitin sulfate and 3% sodium hyaluronate (Viscoat, Alcon Laboratories, Fort Worth, TX, USA), was injected into the anterior chamber. Next, a continuous curvilinear capsulorhexis of 5 to 5.5 mm was created with a forcep. After hydrodissection with balanced salt solution, stop and chop phacoemulsification was performed using the Stellaris phacoemulsification machine (Bausch and Lomb, Rochester, NY, USA). An IOL (Tecnis IOL; Abbott Medical Optics, Santa Ana, CA, USA) was implanted within the capsular bag, and residual viscoelastic material was removed from the anterior chamber. After corneal stromal hydration on the main corneal incision with a balanced salt solution, the anterior chamber was reformed using a balanced salt solution. Postoperative medication was the same for all patients and consisted of topical 1% prednisolone and 0.5% levofloxacin 4 times per day for 4 weeks. Prostaglandin analogs were stopped after surgery for 3 months in the glaucoma patients who were treated with prostaglandin analog before surgery.

### 2.2. Optical Coherence Tomography

The patients underwent Cirrus HD OCT examination preoperatively and postoperatively. For mGC-IPL thickness measurement, the 512 × 128 macular cube was scanned and OCT images were analyzed by the GCA program. To calculate mGC-IPL thickness, the GCA program automatically identifies the outer boundary of the RNFL and the outer boundary of the IPL. The mGC-IPL thickness measurements included average, minimum, and 6 sectoral (superotemporal, superior, superonasal, inferonasal, inferior, and inferotemporal) measures in a macular elliptical annulus (with a vertical inner and outer radius of 0.5 mm and 2.0 mm and a horizontal inner and outer radius of 0.6 mm and 2.4 mm, respectively). OCT examinations were repeated postoperatively at 1 month and 3 months. We manually excluded images with signal strength (SS) < 5 or with segmentation error. According to the manufacturer's recommendation, low SS was defined as < 6.

### 2.3. Statistical Analysis

IBM SPSS ver. 20.0 (IBM Corp., Armonk, NY, USA) was used for all statistical analysis. An independent *t*-test was used to compare continuous variables, and Fisher's exact test was performed to compare categorical variables between groups. The paired *t*-test was performed to compare the mGC-IPL thickness at baseline and postoperatively. The repeated-measure analysis of variance was used to analyze the differences of change in mGC-IPL thickness between the two groups at each time point. Univariate linear regression was used to analyze various factors associated with a postoperative increase in average mGC-IPL thickness. Then, the factors with significance of *P* < 0.2 in the univariate analysis were included in the multivariate regression analysis model. *P*-values less than 0.05 were considered to be statistically significant.

## 3. Results

The mean age of glaucoma and normal group was 76.1 ± 7.8 and 72.5 ± 5.9 years old, respectively. There was no significant difference between the two groups in terms of age, mean preoperative IOP, central corneal thickness, and AXL.  Table 1 displays the subjects' preoperative clinical characteristics. Prostaglandin analog was stopped in four eyes of glaucoma patients after surgery during the study periods.

Table 2 illustrates the changes of mGC-IPL thickness after cataract surgery. The preoperative mean mGC-IPL thickness of glaucoma eyes (69.8 ± 8.2 *µ*m) was significantly thinner than that of normal eyes (74.6 ± 7.2 *µ*m;* P* = 0.043). The mean mGC-IPL thickness was also significantly thinner in glaucoma eyes than in normal eyes at 1 month and 3 months postoperatively (*P* = 0.002 and* P* = 0.001, resp.). The average increase in mGC-IPL thickness showed no significant difference between the two groups. However, in the sectoral analysis, there were significant differences in postoperative change of mGC-IPL thickness in the inferior sector and the inferotemporal sector between the two groups (*P* = 0.004 and* P* = 0.030, resp.). In glaucoma group, inferior mGC-IPL thickness at each time point and inferotemporal mGC-IPL thickness at 3 months after surgery showed no significant increase. The other sectors of both groups increased significantly at postoperative 1 month and 3 months. Comparison of mGC-IPL was made 1 and 3 months after surgery to preoperative values. The average mGC-IPL thickness was not significantly different between 1 and 3 months after surgery (Table 2).

Table 3 presents an analysis of the differences in increased mGC-IPL thickness between the two groups. One month postoperatively, the mean mGC-IPL thickness change was 6.3 ± 7.1% in glaucoma eyes and 8.2 ± 9.5% in normal eyes. Three months postoperatively, it was 5.0 ± 5.6% in glaucoma eyes and 7.5 ± 10.0% in normal eyes. There was a significant difference in postoperative increase of mGC-IPL thickness in the inferior sector between the two groups.

Table 4 indicates the change of SS in OCT images. The SS value increased significantly in normal eyes both 1 month and 3 months postoperatively. In glaucoma eyes, however, there was no significant difference between preoperative and postoperative SS values. SS change showed no significant difference between the two groups (*P* = 0.149).

Table 5 shows the factors associated with the postoperative increased rate of average mGC-IPL thickness. In the multivariate regression analysis, preoperative mGC-IPL thickness showed a significantly negative association with the increased rate of average mGC-IPL thickness postoperatively at 1 month and 3 months (all* P* values < 0.001). In the subgroup analysis, the preoperative mGC-IPL thickness was significantly associated with a postoperative increased rate of average mGC-IPL thickness in glaucoma eyes (Table 6). In normal eyes, AXL and preoperative mGC-IPL thickness were factors associated with a postoperative increased rate of average mGC-IPL thickness 3 months following surgery (Table 7). The postoperative difference in average mGC-IPL thickness was also significantly associated with AXL and preoperative mGC-IPL thickness (Supplementary Tables  1–3) (see Supplementary Material available online at http://dx.doi.org/10.1155/2016/9785939).

## 4. Discussion

In the present study, the mean mGC-IPL thickness was increased 1 month following cataract surgery; at 3 months, it was slightly decreased, but still thicker than the preoperative value, in both normal and glaucoma eyes. Preoperative mGC-IPL thickness and AXL were factors associated with the mGC-IPL thickness change. As far as we know, this study is the first to compare the postoperative change of mGC-IPL thickness between glaucoma and normal eyes.

Our finding of a significant increase in mean mGC-IPL thickness 1 month after cataract surgery is consistent with previous studies. Sari et al. [15] reported that mGC-IPL thickness significantly increased at 1 week and 1 month after cataract surgery. The authors suggested that surgically induced inflammation may affect ganglion cells, resulting in a postoperative increase in mGC-IPL thickness. They showed that the postoperative mGC-IPL thickness tended to return to preoperative values 3 months after surgery. We also found that the postoperative mGC-IPL thickness had a tendency to decrease at 3 months postoperatively, but it was still significantly thicker than preoperative values.

Another possible explanation for the increase of mGC-IPL after cataract surgery is a preoperative measurement error resulting from low SS. Nakatani et al. [12] reported that all parameters of GCC thickness significantly increased 7 weeks after cataract surgery. They suggested that segmentation errors caused by cataract could affect GCC thickness measurements. In the present study, the average SS value increased significantly after surgery. Although we excluded all images with segmentation error manually, the postoperative SS change may have affected the increased mGC-IPL thickness measurement.

Our result showed no significant difference in postoperative change of the mean mGC-IPL thickness between glaucomatous and normal eyes. However, a sectoral analysis indicated that the mGC-IPL thickness of inferior and inferotemporal sector significantly differed between groups. The inferior sector of mGC-IPL thickness in glaucoma patients was not significantly increased at 1 month and 3 months after cataract surgery. The inferotemporal sector thickness in glaucoma patients also showed no significant change 3 months after surgery. Studies indicate that the inferior and inferotemporal sector are the most common sites of glaucomatous damage [16, 17]. Aligning with previous studies, our results show that the preoperative mGC-IPL thickness of the inferior and inferotemporal sector was thinner compared to other sectors. The effect of SS, which may cause underestimation of preoperative mGC-IPL thickness, would be similar for all sectors in one subject. Taken together, these results suggest that the postoperative increase of mGC-IPL thickness could be affected by postoperative inflammation more than SS change.

The only available report comparing changes of GCC thickness in glaucoma eyes with normal eyes after cataract surgery showed that the presence of glaucoma correlated with greater changes in postoperative GCC thickness [12]. In that study, the difference of average GCC thickness was 5.6 ± 6.4 um in glaucoma eyes and 1.4 ± 2.8 um in normal eyes. We also compared the differences of change in mGC-IPL thickness between two groups 1 month and 3 months postoperatively. In contrast to the previous report, we found a similar thickness change on average between the two groups. Compared with preoperative values, the mean postoperative increase of mGC-IPL thickness did not significantly differ between glaucoma eyes and normal eyes at any follow-up visit. This discrepancy can be explained by different types of OCT (RTVue versus Cirrus) and the small number of glaucoma patients in the previous study (*n* = 7). Further studies will be needed to determine the association between glaucoma and postoperative changes in mGC-IPL thickness.

We demonstrated the mGC-IPL thickness changes showed a negative correlation with preoperative mGC-IPL thickness. In the previous analysis, the more damaged sectors of glaucoma eyes, thinner than other sectors, showed less change in mGC-IPL thickness. However, the linear regression analysis revealed that the mGC-IPL thickness changes had a negative association with preoperative mGC-IPL thickness, even in glaucoma patients (Table 6). The postulated reason for this finding is that the thin retina may be vulnerable to breakdown of the blood-retina membrane after surgery, possibly resulting in more changes in mGC-IPL thickness. Recently, Sacchi et al. [18] evaluated macular thickness changes after pediatric cataract surgery using spectral domain OCT. Similar to our results, they reported that macular thickness remained increased for more than 3 months after surgery, but no eye showed cystoid macular edema. They indicated that the healthy vasculature and intact blood-retinal membranes of young patients influenced their results. Further studies are needed to confirm our findings.

Additionally, AXL showed a negative correlation with postoperative increased rate of average mGC-IPL thickness in normal eyes. Several studies reported that AXL was negatively associated with GCC thickness in normal eyes [19, 20]. Regarding our results, we speculate that retinal layers in eyes with longer AXL that were tangentially stretched by globe elongation may have little capacity to increase after cataract surgery. Another explanation could rest in magnification error, which may cause underestimation not only in the GCC thickness but also in the mGC-IPL thickness changes.

The main limitation of present study is the small number of glaucoma eyes. Because of the retrospective design of this study, we were unable to include large number of glaucoma patients. Moreover, the follow-up period was relatively short. Further prospective studies with a large number of patients should be performed to analyze the long-term effects of cataract surgery on mGC-IPL thickness

In conclusion, the mean mGC-IPL thickness was increased up to 3 months after cataract surgery, and the postoperative changes of mGC-IPL thickness were associated with preoperative mGC-IPL thickness in both groups and AXL in normal eyes. The effects of cataract surgery on mean mGC-IPL thickness change were not different in glaucomatous and normal eyes. However, more damaged sectors of glaucomatous eyes showed less change in terms of mGC-IPL thickness in the sectoral analysis. Further studies will be needed to validate the effect of cataract surgery on mGC-IPL thickness in glaucoma patients.

## Supplementary Material

Supplementary Tables shows the factors associated with the postoperative difference in average mGC-IPL thickness postoperatively at 1 month and 3 months. The preoperative average mGC-IPL thickness was significantly associated with the postoperative difference in average mGC-IPL thickness at 3 months after surgery (Supplementary Table 1). In the subgroup analysis, the postoperative difference in average mGC-IPL thickness was significantly associated with a preoperative mGC-IPL thickness in glaucoma eyes (Supplementary Table 2). In normal eyes, AXL and preoperative average mGC-IPL thickness were associated with the postoperative difference in average mGC-IPL thickness (supplementary Table 3).

## Figures and Tables

**Table 1:** Preoperative characteristics of study participants.

	Glaucoma	Normal	*P* value
	(*N* = 13)	(*N* = 52)	
Age (years)	76.1 ± 7.8	72.5 ± 5.9	0.074^a^
Sex (M/F)	2/11	19/33	0.194^b^
BCVA (logMAR*⁡*)	0.3 ± 0.3	0.2 ± 0.2	0.107^a^
IOP (mmHg)	12.5 ± 2.8	12.0 ± 2.5	0.576^a^
CCT (*µ*m)	516.6 ± 43.1	530.1 ± 26.4	0.298^a^
AXL (mm)	23.4 ± 0.9	23.6 ± 0.9	0.396^a^

Values are presented as mean ± standard deviation or number.

BCVA: best-corrected visual acuity; logMAR: logarithm of the minimum angle of resolution; IOP: intraocular pressure; CCT: central corneal thickness; AXL: axial length.

^a^Independent  *t*-test.

^b^Fisher's exact test.

**Table 2:** Pre- and postoperative average and sectoral macular ganglion cell-inner plexiform layer thickness in glaucoma and normal eyes.

Parameters (*µ*m)	Preoperative	1 month	*P* value^a^	3 months	*P *value^a^	*P *value^b^
Average						
Glaucoma	69.8 ± 8.2	73.8 ± 6.4	0.001	73.0 ± 6.4	0.001	0.171
Normal	74.6 ± 7.2	80.3 ± 6.7	<0.001	79.8 ± 6.5	<0.001	
*P *value^c^	0.043	0.002		0.001		
Superior						
Glaucoma	70.8 ± 13.4	76.2 ± 9.2	0.008	75.2 ± 9.5	0.013	0.791
Normal	75.0 ± 9.6	80.7 ± 7.9	<0.001	80.3 ± 7.8	<0.001	
Superonasal						
Glaucoma	73.2 ± 13.1	77.5 ± 11.2	0.001	76.9 ± 12.0	<0.001	0.803
Normal	76.6 ± 9.0	81.8 ± 9.5	<0.001	80.9 ± 10.3	0.002	
Inferonasal						
Glaucoma	70.1 ± 12.9	75.3 ± 8.7	0.012	74.5 ± 8.3	0.017	0.936
Normal	74.3 ± 8.4	79.8 ± 7.8	<0.001	79.0 ± 8.3	<0.001	
Inferior						
Glaucoma	68.1 ± 8.9	69.1 ± 9.6	0.513	68.8 ± 9.0	0.542	0.004
Normal	72.6 ± 7.2	77.6 ± 6.7	<0.001	77.2 ± 7.1	<0.001	
Inferotemporal						
Glaucoma	67.5 ± 9.8	70.5 ± 8.9	0.002	69.3 ± 9.1	0.134	0.030
Normal	75.7 ± 7.4	81.3 ± 7.9	<0.001	81.3 ± 7.8	<0.001	
Superotemporal						
Glaucoma	69.8 ± 7.1	74.6 ± 6.7	<0.001	73.8 ± 6.5	<0.001	0.438
Normal	74.2 ± 9.6	80.5± 7.6	<0.001	80.0 ± 7.9	<0.001	

Values are presented as mean ± standard deviation.

^a^Paired*  t*-test.

^b^Repeated-measure analysis of variance.

^c^Independent*  t*-test.

**Table 3:** Comparison of average and sectoral macular ganglion cell-inner plexiform layer thickness change after cataract surgery between glaucoma and normal eyes.

Parameters (*µ*m)	1 month		3 months	
	Difference	Increase rate (%)	Difference	Increase rate (%)
Average				
Glaucoma	3.9 ± 3.1	6.3 ± 7.1	3.2 ± 2.5	5.0 ± 5.6
Normal	5.7 ± 4.5	8.2 ± 9.5	5.2 ± 4.8	7.5 ± 10.0
*P* value	0.180	0.503	0.142	0.400
Superior				
Glaucoma	5.5 ± 6.2	10.9 ± 21.1	4.5 ± 5.5	9.2 ± 18.7
Normal	5.7 ± 6.3	9.8 ± 24.4	5.3 ± 6.7	9.4 ± 25.1
*P* value	0.891	0.882	0.669	0.976
Superonasal				
Glaucoma	4.3 ± 3.8	7.3 ± 10.6	3.7 ± 2.8	6.1 ± 8.2
Normal	5.2 ± 8.6	8.2 ± 19.8	4.3 ± 9.6	7.0 ± 21.6
*P* value	0.714	0.887	0.821	0.879
Inferonasal				
Glaucoma	5.2 ± 6.4	10.7 ± 21.2	4.4 ± 5.7	9.2 ± 18.8
Normal	5.5 ± 6.6	8.1 ± 11.2	4.7 ± 7.0	7.0 ± 11.5
*P* value	0.910	0.542	0.884	0.594
Inferior				
Glaucoma	1.0 ± 5.4	1.7 ± 8.4	0.8 ± 4.4	1.3 ± 6.8
Normal	5.0 ± 4.4	7.2 ± 7.0	4.6 ± 4.5	6.6 ± 7.0
*P* value	0.006	0.017	0.007	0.017
Inferotemporal				
Glaucoma	3.0 ± 2.7	4.8 ± 4.7	1.8 ± 4.1	3.1 ± 6.3
Normal	5.6 ± 4.9	7.6 ± 8.1	5.6 ± 5.3	7.7 ± 8.9
*P* value	0.073	0.237	0.022	0.085
Superotemporal				
Glaucoma	4.8 ± 1.8	7.0 ± 2.9	3.9 ± 1.7	5.8 ± 2.9
Normal	6.4 ± 7.0	11.0 ± 26.1	5.8 ± 7.5	10.2 ± 26.4
*P* value	0.420	0.586	0.372	0.551

Values are presented as mean ± standard deviation.

**Table 4:** Pre- and postoperative signal strength of optical coherence tomography images in glaucoma and normal eyes.

	Preoperative	1 month	*P *value^a^	3 months	*P *value^a^	*P *value^b^
Average	6.9 ± 1.1	8.2 ± 1.3	<0.001	7.9 ± 1.2	<0.001	
Glaucoma	6.5 ± 1.3	7.5 ± 1.6	0.084	6.8 ± 1.1	0.356	0.149
Normal	7.0 ± 1.0	8.4 ± 1.1	<0.001	8.2 ± 1.1	<0.001	

Values are presented as mean ± standard deviation.

^a^Paired*  t*-test.

^b^Repeated-measure analysis of variance.

**Table 5:** Univariate and multivariate analysis of factors associated with postoperative increase rate of average macular ganglion cell-inner plexiform layer thickness in glaucoma and normal eyes.

Parameters	1 month				3 months			
	Univariate		Multivariate		Univariate		Multivariate	
	Regression coefficient	*P* value	Regression coefficient	*P* value	Regression coefficient	*P* value	Regression coefficient	*P* value
Age (years)	0.196	0.272			0.202	0.267		
Preoperative BCVA (logMAR)	−0.224	0.969			−0.708	0.905		
Preoperative IOP	0.643	0.154	0.326	0.406	0.711	0.122	0.538	0.170
Preoperative SS	−1.052	0.317			−1.056	0.325		
AXL	−1.097	0.386			−1.371	0.288		
CCT	0.061	0.104	0.033	0.313	0.045	0.236		
Preoperative average mGC-IPL thickness	−0.638	<0.001	−0.600	<0.001	−0.652	<0.001	−0.639	<0.001
IOP change	−0.320	0.518			−0.112	0.829		
SS change	0.594	0.400			1.106	0.162	1.410	0.036

BCVA: best-corrected visual acuity; logMAR: logarithm of the minimum angle of resolution; IOP: intraocular pressure; SS: signal strength; AXL: axial length; CCT: central corneal thicken; mGC-IPL: macular ganglion cell-inner plexiform layer.

**Table 6:** Univariate and multivariate analysis of factors associated with postoperative increase rate of average macular ganglion cell-inner plexiform layer thickness in glaucoma eyes.

Parameters	1 month				3 months			
	Univariate		Multivariate		Univariate		Multivariate	
	Regression coefficient	*P* value	Regression coefficient	*P* value	Regression coefficient	*P* value	Regression coefficient	*P* value
Age (years)	0.536	0.034	0.257	0.321	0.301	0.148	−0.009	0.956
Preoperative BCVA (logMAR)	−3.162	0.702			−5.036	0.430		
Preoperative IOP	0.520	0.505			0.452	0.456		
Preoperative SS	−2.399	0.146	0.232	0.894	−1.707	0.189	0.534	0.651
AXL	−0.211	0.930			1.009	0.587		
CCT	0.075	0.121	−0.018	0.707	0.053	0.159	−0.004	0.910
Preoperative average mGC-IPL thickness	−0.721	<0.001	−0.728	0.018	−0.596	<0.001	−0.690	0.006
IOP change	0.748	0.472			−0.041	0.969		
SS change	1.414	0.165	-0.481	0.643	2.099	0.053	−0.259	0.823

BCVA: best-corrected visual acuity; logMAR: logarithm of the minimum angle of resolution; IOP: intraocular pressure; SS: signal strength; AXL: axial length; CCT: central corneal thicken; mGC-IPL: macular ganglion cell-inner plexiform layer.

**Table 7:** Univariate and multivariate analysis of factors associated with postoperative increase rate of average macular ganglion cell-inner plexiform layer thickness in normal eyes.

Parameters	1 month				3 months			
	Univariate		Multivariate		Univariate		Multivariate	
	Regression coefficient	*P* value	Regression coefficient	*P* value	Regression coefficient	*P* value	Regression coefficient	*P* value
Age (years)	0.108	0.639			0.227	0.345		
Preoperative BCVA (logMAR)	2.617	0.736			3.212	0.693		
Preoperative IOP	0.712	0.189	0.394	0.403	0.828	0.143	−0.063	0.900
Preoperative SS	−0.806	0.540			−1.110	0.419		
AXL	−1.439	0.334			−2.108	0.174	−3.575	0.012
CCT	0.047	0.354			0.033	0.540		
Preoperative average mGC-IPL thickness	−0.704	<0.001	−0.683	<0.001	−0.772	<0.001	−0.867	<0.001
IOP change	−0.520	0.358			−0.190	0.748		
SS change	0.185	0.836			0.744	0.448		

BCVA: best-corrected visual acuity; logMAR: logarithm of the minimum angle of resolution; IOP: intraocular pressure; SS: signal strength; AXL: axial length; CCT: central corneal thicken; mGC-IPL: macular ganglion cell-inner plexiform layer.
