# Supplementary material for: Changes of the Macular Ganglion Cell-Inner Plexiform Layer Thickness after Cataract Surgery in Glaucoma Patients
Source: J Ophthalmol. 2016 Dec 22;2016:9785939. doi: 10.1155/2016/9785939 (PMC5214588; doi:10.1155/2016/9785939)
Supplement: Supplementary file 1 — Supplementary Tables shows the factors associated with the postoperative difference in average mGC-IPL thickness postoperatively at 1 month and 3 months. The preoperative average mGC-IPL thickness was significantly associated with the postoperative difference in average mGC-IPL thickness at 3 months after surgery (Supplementary Table 1). In the subgroup analysis, the postoperative difference in average mGC-IPL thickness was significantly associated with a preoperative mGC-IPL thickness in glaucoma eyes (Supplementary Table 2). In normal eyes, AXL and preoperative average mGC-IPL thickness were associated with the postoperative difference in average mGC-IPL thickness (supplementary Table 3). [file 9785939.f1.docx]

**Supplementary Table 1.** Univariate and multivariate analysis of factors associated with postoperative difference in average macular ganglion cell-inner plexiform layer thickness in glaucoma and normal eyes

|  | 1 month | | | | 3 months | | | |
| --- | --- | --- | --- | --- | --- | --- | --- | --- |
| Parameters | Univariate | | Multivariate | | Univariate | | Multivariate | |
|  | Regression coefficient | *P* value | Regression coefficient | *P* value | Regression coefficient | *P* value | Regression coefficient | *P* value |
| Age (years) | 0.033 | 0.698 |  |  | 0.056 | 0.526 |  |  |
| Preoperative BCVA (logMAR) | -0.841 | 0.762 |  |  | -1.224 | 0.669 |  |  |
| Preoperative IOP | 0.239 | 0.269 |  |  | 0.293 | 0.188 | 0.125 | 0.531 |
| Preoperative SS | -0.326 | 0.517 |  |  | -0.369 | 0.477 |  |  |
| AXL | -0.740 | 0.218 |  |  | -0.938 | 0.129 | -1.387 | 0.015 |
| CCT | 0.029 | 0.099 | 0.021 | 0.216 | 0.019 | 0.307 |  |  |
| Preoperative average mGC-IPL thickness | -0.221 | 0.001 | -0.208 | 0.003 | -0.247 | <0.001 | -0.280 | 0.015 |
| IOP change | -0.264 | 0.262 |  |  | -0.094 | 0.707 |  |  |
| SS change | 0.302 | 0.369 |  |  | 0.650 | 0.087 | 0.816 | 0.015 |

BCVA, best-corrected visual acuity; logMAR, logarithm of the minimum angle of resolution; IOP, intraocular pressure; SS, signal strength; AXL, axial length; CCT, central corneal thicken; mGC-IPL, macular ganglion cell-inner plexiform layer.

**Supplementary Table 2**. Univariate and multivariate analysis of factors associated with postoperative difference in average macular ganglion cell-inner plexiform layer thickness in glaucoma eyes

|  | 1 month | | | | 3 months | | | |
| --- | --- | --- | --- | --- | --- | --- | --- | --- |
| Parameters | Univariate | | Multivariate | | Univariate | | Multivariate | |
|  | Regression coefficient | *P* value | Regression coefficient | *P* value | Regression coefficient | *P* value | Regression coefficient | *P* value |
| Age (years) | 0.242 | 0.030 | 0.135 | 0.313 | 0.110 | 0.252 |  |  |
| Preoperative BCVA (logMAR) | -1.284 | 0.726 |  |  | -2.800 | 0.328 |  |  |
| Preoperative IOP | 0.262 | 0.445 |  |  | 0.237 | 0.386 |  |  |
| Preoperative SS | -1.068 | 0.143 | 0.107 | 0.890 | -0.724 | 0.220 |  |  |
| AXL | -0.506 | 0.632 |  |  | 0.414 | 0.623 |  |  |
| CCT | 0.033 | 0.124 | -0.003 | 0.910 | 0.022 | 0.197 | -0.002 | 0.864 |
| Preoperative average mGC-IPL thickness | -0.272 | 0.007 | -0.219 | 0.100 | -0.239 | 0.002 | -0.272 | 0.019 |
| IOP change | 0.291 | 0.527 |  |  | -0.183 | 0.705 |  |  |
| SS change | 0.532 | 0.244 |  |  | 0.847 | 0.091 | -0.212 | 0.683 |

BCVA, best-corrected visual acuity; logMAR, logarithm of the minimum angle of resolution; IOP, intraocular pressure; SS, signal strength; AXL, axial length; CCT, central corneal thicken; mGC-IPL, macular ganglion cell-inner plexiform layer.

**Supplementary Table 3**. Univariate and multivariate analysis of factors associated with postoperative difference in average macular ganglion cell-inner plexiform layer thickness in normal eyes

|  | 1 month | | | | 3 months | | | |
| --- | --- | --- | --- | --- | --- | --- | --- | --- |
| Parameters | Univariate | | Multivariate | | Univariate | | Multivariate | |
|  | Regression coefficient | *P* value | Regression coefficient | *P* value | Regression coefficient | *P* value | Regression coefficient | *P* value |
| Age (years) | -0.014 | 0.899 |  |  | 0.080 | 0.487 |  |  |
| Preoperative BCVA (logMAR) | 0.543 | 0.883 |  |  | 0.902 | 0.816 |  |  |
| Preoperative IOP | 0.260 | 0.315 |  |  | 0.341 | 0.208 |  |  |
| Preoperative SS | -0.251 | 0.688 |  |  | -0.456 | 0.487 |  |  |
| AXL | -0.915 | 0.194 | -1.210 | 0.071 | -1.398 | 0.057 | -1.981 | 0.002 |
| CCT | 0.022 | 0.376 |  |  | 0.010 | 0.709 |  |  |
| Preoperative average mGC-IPL thickness | -0.259 | 0.002 | -0.292 | 0.001 | -0.309 | <0.001 | -0.360 | <0.001 |
| IOP change | -0.371 | 0.165 | -0.269 | 0.274 | -0.143 | 0.612 |  |  |
| SS change | 0.152 | 0.722 |  |  | 0.478 | 0.307 |  |  |

BCVA, best-corrected visual acuity; logMAR, logarithm of the minimum angle of resolution; IOP, intraocular pressure; SS, signal strength; AXL, axial length; CCT, central corneal thicken; mGC-IPL, macular ganglion cell-inner plexiform layer.
